# Supplementary material for: Visual Affective Stimulus Database: A Validated Set of Short Videos
Source: Behav Sci (Basel). 2022 May 9;12(5):137. doi: 10.3390/bs12050137 (PMC9138138; doi:10.3390/bs12050137)
Supplement: Supplementary file 1 [file behavsci-12-00137-s001.zip › behavsci-1686428-supplementary.pdf]

Visual Affective Stimulus Database: A Validated Set of Short Videos

Supplementary Materials

Table S1. Recognition rate for valence of videos

	N	Min（%）	Max（%）	Mean（%）
Positive	115	60.3	89.7	72.9
Neutral	52	60.3	89.7	73.8
Negative	83	60.3	96.5	80.8

Table S2. The percentage of the recognition rate of all the videos

Number	Valence > 5 (%)	Valence = 5 (%)	Valence ＜ 5 (%)
1	73.28%	25.00%	1.72%
2	81.90%	16.38%	1.72%
3	81.90%	17.24%	0.86%
4	63.79%	35.34%	0.86%
5	62.93%	32.76%	4.31%
6	77.59%	17.24%	5.17%
7	53.45%	31.03%	15.52%
8	62.07%	35.34%	2.59%
9	56.03%	42.24%	1.72%
10	61.21%	38.79%	0.00%
11	43.97%	44.83%	11.21%
12	62.07%	35.34%	2.59%
13	65.52%	33.62%	0.86%
14	77.59%	19.83%	2.59%
15	80.17%	17.24%	2.59%
16	61.21%	36.21%	2.59%
17	45.69%	50.00%	4.31%
18	50.86%	45.69%	3.45%
19	52.59%	44.83%	2.59%
20	56.03%	39.66%	4.31%
21	76.72%	19.83%	3.45%
22	76.72%	23.28%	0.00%
23	71.55%	26.72%	1.72%
24	65.52%	28.45%	6.03%
25	75.86%	18.97%	5.17%
26	78.45%	20.69%	0.86%
27	75.00%	25.00%	0.00%
28	81.90%	15.52%	2.59%
29	70.69%	24.14%	5.17%
30	78.45%	19.83%	1.72%
31	76.72%	18.10%	5.17%
32	72.41%	25.00%	2.59%
33	81.03%	18.10%	0.86%
34	50.86%	43.97%	5.17%
35	68.10%	29.31%	2.59%
36	75.00%	19.83%	5.17%
37	51.72%	46.55%	1.72%
38	66.38%	26.72%	6.90%

39	50.86%	43.97%	5.17%
40	68.97%	31.03%	0.00%
41	65.52%	34.48%	0.00%
42	70.69%	28.45%	0.86%
43	68.97%	30.17%	0.86%
44	64.66%	32.76%	2.59%
45	57.76%	38.79%	3.45%
46	63.79%	35.34%	0.86%
47	68.10%	28.45%	3.45%
48	73.28%	24.14%	2.59%
49	71.55%	24.14%	4.31%
50	71.55%	24.14%	4.31%
51	80.17%	18.10%	1.72%
52	75.00%	23.28%	1.72%
53	71.55%	25.00%	3.45%
54	72.41%	25.00%	2.59%
55	52.59%	43.10%	4.31%
56	74.14%	23.28%	2.59%
57	72.41%	24.14%	3.45%
58	61.21%	38.79%	0.00%
59	62.93%	35.34%	1.72%
60	80.17%	17.24%	2.59%
61	62.93%	35.34%	1.72%
62	81.90%	16.38%	1.72%
63	83.62%	14.66%	1.72%
64	56.90%	37.93%	5.17%
65	74.14%	23.28%	2.59%
66	55.17%	41.38%	3.45%
67	67.24%	26.72%	6.03%
68	75.86%	21.55%	2.59%
69	76.72%	18.97%	4.31%
70	81.90%	14.66%	3.45%
71	74.14%	23.28%	2.59%
72	68.97%	27.59%	3.45%
73	70.69%	26.72%	2.59%
74	74.14%	23.28%	2.59%
75	75.86%	22.41%	1.72%
76	75.86%	21.55%	2.59%
77	71.55%	20.69%	7.76%
78	71.55%	24.14%	4.31%
79	75.86%	22.41%	1.72%
80	70.69%	21.55%	7.76%
81	63.79%	34.48%	1.72%
82	69.83%	27.59%	2.59%

83	62.93%	28.45%	8.62%
84	64.66%	19.83%	15.52%
85	69.83%	25.86%	4.31%
86	68.97%	31.03%	0.00%
87	62.93%	32.76%	4.31%
88	67.24%	30.17%	2.59%
89	56.90%	39.66%	3.45%
90	72.41%	27.59%	0.00%
91	73.28%	25.86%	0.86%
92	66.38%	33.62%	0.00%
93	70.69%	25.00%	4.31%
94	73.28%	25.86%	0.86%
95	70.69%	28.45%	0.86%
96	63.79%	34.48%	1.72%
97	59.48%	24.14%	16.38%
98	74.14%	23.28%	2.59%
99	73.28%	25.00%	1.72%
100	78.45%	18.97%	2.59%
101	76.72%	20.69%	2.59%
102	71.55%	22.41%	6.03%
103	73.28%	22.41%	4.31%
104	56.03%	37.93%	6.03%
105	75.86%	23.28%	0.86%
106	74.14%	25.00%	0.86%
107	74.14%	18.97%	6.90%
108	77.59%	21.55%	0.86%
109	74.14%	21.55%	4.31%
110	81.03%	18.10%	0.86%
111	81.90%	16.38%	1.72%
112	83.62%	13.79%	2.59%
113	89.66%	7.76%	2.59%
114	81.03%	15.52%	3.45%
115	81.90%	13.79%	4.31%
116	70.69%	24.14%	5.17%
117	68.10%	26.72%	5.17%
118	82.76%	14.66%	2.59%
119	73.28%	19.83%	6.90%
120	78.45%	16.38%	5.17%
121	74.14%	23.28%	2.59%
122	72.41%	25.86%	1.72%
123	73.28%	25.00%	1.72%
124	81.03%	16.38%	2.59%
125	66.38%	24.14%	9.48%
126	83.62%	14.66%	1.72%

127	81.03%	15.52%	3.45%
128	81.03%	17.24%	1.72%
129	85.34%	13.79%	0.86%
130	53.45%	33.62%	12.93%
131	40.52%	51.72%	7.76%
132	34.48%	60.34%	5.17%
133	22.41%	74.14%	3.45%
134	21.55%	75.86%	2.59%
135	31.90%	65.52%	2.59%
136	33.62%	62.07%	4.31%
137	16.38%	75.00%	8.62%
138	25.86%	69.83%	4.31%
139	21.55%	70.69%	7.76%
140	9.48%	84.48%	6.03%
141	9.48%	86.21%	4.31%
142	12.93%	77.59%	9.48%
143	25.86%	68.97%	5.17%
144	26.72%	70.69%	2.59%
145	22.41%	75.00%	2.59%
146	17.24%	80.17%	2.59%
147	11.21%	72.41%	16.38%
148	43.10%	50.00%	6.90%
149	20.69%	64.66%	14.66%
150	53.45%	43.97%	2.59%
151	33.62%	51.72%	14.66%
152	14.66%	73.28%	12.07%
153	54.31%	42.24%	3.45%
154	44.83%	50.00%	5.17%
155	37.93%	55.17%	6.90%
156	28.45%	68.10%	3.45%
157	40.52%	51.72%	7.76%
158	37.93%	56.90%	5.17%
159	32.76%	58.62%	8.62%
160	59.48%	36.21%	4.31%
161	67.24%	32.76%	0.00%
162	45.69%	50.00%	4.31%
163	60.34%	37.93%	1.72%
164	68.97%	30.17%	0.86%
165	54.31%	40.52%	5.17%
166	46.55%	50.00%	3.45%
167	27.59%	70.69%	1.72%
168	25.00%	62.07%	12.93%
169	50.86%	43.97%	5.17%
170	39.66%	57.76%	2.59%

171	58.62%	38.79%	2.59%
172	14.66%	81.90%	3.45%
173	12.93%	77.59%	9.48%
174	43.10%	54.31%	2.59%
175	12.93%	77.59%	9.48%
176	14.66%	82.76%	2.59%
177	7.76%	89.66%	2.59%
178	12.93%	82.76%	4.31%
179	13.79%	83.62%	2.59%
180	15.52%	81.90%	2.59%
181	29.31%	64.66%	6.03%
182	16.38%	81.03%	2.59%
183	10.34%	84.48%	5.17%
184	16.38%	81.03%	2.59%
185	26.72%	69.83%	3.45%
186	30.17%	67.24%	2.59%
187	34.48%	62.93%	2.59%
188	43.97%	53.45%	2.59%
189	50.00%	45.69%	4.31%
190	37.07%	59.48%	3.45%
191	15.52%	67.24%	17.24%
192	27.59%	68.97%	3.45%
193	3.45%	73.28%	23.28%
194	5.17%	82.76%	12.07%
195	24.14%	75.00%	0.86%
196	16.38%	75.86%	7.76%
197	22.41%	73.28%	4.31%
198	37.93%	60.34%	1.72%
199	16.38%	75.00%	8.62%
200	31.03%	65.52%	3.45%
201	16.38%	79.31%	4.31%
202	28.45%	57.76%	13.79%
203	25.86%	59.48%	14.66%
204	4.31%	12.93%	82.76%
205	7.76%	25.00%	67.24%
206	1.72%	7.76%	90.52%
207	0.86%	10.34%	88.79%
208	0.00%	15.52%	84.48%
209	0.86%	8.62%	90.52%
210	1.72%	6.90%	91.38%
211	1.72%	11.21%	87.07%
212	6.03%	18.10%	75.86%
213	8.62%	23.28%	68.10%
214	2.59%	12.93%	84.48%

215	2.59%	18.10%	79.31%
216	3.45%	53.45%	43.10%
217	8.62%	59.48%	31.90%
218	6.03%	70.69%	23.28%
219	14.66%	35.34%	50.00%
220	23.28%	29.31%	47.41%
221	6.03%	11.21%	82.76%
222	6.03%	12.93%	81.03%
223	6.03%	12.07%	81.90%
224	3.45%	11.21%	85.34%
225	4.31%	12.93%	82.76%
226	1.72%	15.52%	82.76%
227	1.72%	16.38%	81.90%
228	1.72%	7.76%	90.52%
229	3.45%	12.93%	83.62%
230	2.59%	25.86%	71.55%
231	1.72%	24.14%	74.14%
232	3.45%	16.38%	80.17%
233	2.59%	37.93%	59.48%
234	1.72%	16.38%	81.90%
235	6.03%	44.83%	49.14%
236	3.45%	13.79%	82.76%
237	1.72%	10.34%	87.93%
238	3.45%	75.86%	20.69%
239	6.90%	67.24%	25.86%
240	9.48%	66.38%	24.14%
241	6.03%	83.62%	10.34%
242	0.86%	19.83%	79.31%
243	1.72%	16.38%	81.90%
244	5.17%	18.97%	75.86%
245	0.00%	14.66%	85.34%
246	2.59%	12.07%	85.34%
247	1.72%	9.48%	88.79%
248	6.90%	29.31%	63.79%
249	2.59%	16.38%	81.03%
250	2.59%	18.97%	78.45%
251	6.03%	15.52%	78.45%
252	4.31%	24.14%	71.55%
253	3.45%	33.62%	62.93%
254	3.45%	26.72%	69.83%
255	5.17%	22.41%	72.41%
256	1.72%	15.52%	82.76%
257	0.86%	17.24%	81.90%
258	0.86%	14.66%	84.48%

259	1.72%	13.79%	84.48%
260	5.17%	25.00%	69.83%
261	2.59%	16.38%	81.03%
262	2.59%	19.83%	77.59%
263	4.31%	20.69%	75.00%
264	3.45%	20.69%	75.86%
265	3.45%	20.69%	75.86%
266	9.48%	22.41%	68.10%
267	2.59%	13.79%	83.62%
268	2.59%	6.90%	90.52%
269	3.45%	4.31%	92.24%
270	1.72%	16.38%	81.90%
271	0.86%	7.76%	91.38%
272	0.86%	2.59%	96.55%
273	0.00%	4.31%	95.69%
274	6.03%	18.97%	75.00%
275	0.86%	11.21%	87.93%
276	12.07%	27.59%	60.34%
277	2.59%	11.21%	86.21%
278	0.86%	14.66%	84.48%
279	12.93%	22.41%	64.66%
280	0.00%	6.03%	93.97%
281	4.31%	5.17%	90.52%
282	2.59%	4.31%	93.10%
283	7.76%	32.76%	59.48%
284	7.76%	24.14%	68.10%
285	16.38%	12.07%	71.55%
286	3.45%	43.97%	52.59%
287	5.17%	28.45%	66.38%
288	1.72%	10.34%	87.93%
289	6.03%	29.31%	64.66%
290	0.86%	36.21%	62.93%
291	0.86%	10.34%	88.79%
292	1.72%	12.07%	86.21%
293	3.45%	17.24%	79.31%
294	4.31%	4.31%	91.38%
295	0.86%	8.62%	90.52%
296	3.45%	4.31%	92.24%
297	2.59%	15.52%	81.90%
298	3.45%	34.48%	62.07%
299	0.86%	11.21%	87.93%

Table S3. The average recognition scores of each video on three emotion dimensions

Number	ID	valence	Arousal	Dominance
1	1	6.38	5.81	6.27
2	2	6.82	6.59	6.77
3	3	6.72	6.47	6.57
4	4	6.16	5.76	6.12
5	5	6.15	6.01	6.13
6	6	6.43	6.29	6.34
7	12	6.03	5.67	6.04
8	13	6.19	5.88	6.1
9	14	6.43	6.15	6.28
10	15	6.47	6.24	6.42
11	16	5.96	5.58	5.96
12	21	6.54	6.26	6.44
13	22	6.65	6.38	6.6
14	23	6.4	6.2	6.36
15	24	6.07	5.92	6.05
16	25	6.46	6.26	6.49
17	26	6.49	6.21	6.45
18	27	6.59	6.33	6.51
19	28	6.78	6	6.69
20	29	6.29	5.99	6.12
21	30	6.57	6.25	6.45
22	31	6.54	6.28	6.56
23	32	6.35	6.07	6.2
24	33	6.57	6.27	6.47
25	35	6.1	5.8	6.12
26	36	6.44	6.37	6.51
27	38	5.95	5.54	5.85
28	40	6.22	5.8	6.33
29	41	6.03	5.71	6.05
30	42	6.32	5.79	6.27
31	43	6.15	5.77	6
32	47	6.15	5.73	5.99
33	48	6.25	5.87	6.22
34	49	6.1	5.62	5.94
35	50	6.14	5.77	6.06
36	51	6.54	5.99	6.47
37	52	6.13	5.74	6.11
38	53	6.28	5.93	6.25
39	54	6.2	5.86	6.09
40	56	6.35	5.85	6.23

41	57	6.25	5.85	6.2
42	58	5.96	5.66	5.97
43	59	6.11	5.66	6.08
44	60	6.48	6.04	6.46
45	61	6.11	5.61	6.08
46	62	6.88	6.49	6.86
47	63	6.73	6.26	6.62
48	65	6.37	6	6.24
49	67	6.08	5.78	6.12
50	68	6.48	6.02	6.37
51	69	6.19	5.9	6.23
52	70	6.7	6.24	6.52
53	71	6.41	6.09	6.34
54	72	5.99	5.73	6.01
55	73	6.13	5.73	6.05
56	74	6.37	6.12	6.31
57	75	6.29	5.99	6.27
58	76	6.38	6.34	6.39
59	77	6.11	6.16	6.13
60	78	6.21	6.03	6.1
61	79	6.47	6.13	6.33
62	80	6.09	6.09	5.99
63	81	5.9	5.61	5.85
64	82	6.25	5.8	6.18
65	85	6.07	5.77	6.08
66	86	6.33	5.8	6.37
67	88	6.06	5.65	6.1
68	90	6.43	5.85	6.25
69	91	6.25	5.81	6
70	92	6.05	5.66	5.97
71	93	6.18	5.93	6.19
72	94	6.1	5.67	6
73	95	6.05	5.66	5.99
74	96	6.23	5.8	6.06
75	98	6.52	6.36	6.36
76	99	6.49	6.11	6.44
77	100	6.67	6.43	6.7
78	101	6.59	6.23	6.59
79	102	6.33	6.05	6.36
80	103	6.35	5.92	6.22
81	105	6.44	5.99	6.32
82	106	6.42	6.09	6.36
83	107	6.12	6.13	6.13
84	108	6.42	5.89	6.41

85	109	6.39	6.1	6.37
86	110	6.87	6.4	6.75
87	111	6.62	6.21	6.46
88	112	6.91	6.71	6.91
89	113	6.9	6.52	6.67
90	114	6.66	6.56	6.72
91	115	6.85	6.51	6.77
92	116	6.29	6.04	6.27
93	117	6.16	5.89	6.13
94	118	6.75	6.49	6.68
95	119	6.24	5.98	6.11
96	120	6.38	6.3	6.31
97	121	6.38	6.22	6.36
98	122	6.37	6.08	6.32
99	123	6.43	6.2	6.41
100	124	6.82	6.69	6.87
101	125	6.07	6.16	5.89
102	126	7	6.74	6.93
103	127	6.87	6.75	6.87
104	128	6.6	6.27	6.66
105	129	6.95	6.27	6.95
106	132	5.41	4.95	5.45
107	133	5.34	5.25	5.54
108	134	5.33	5.18	5.36
109	135	5.45	5.23	5.44
110	136	5.4	5.28	5.4
111	137	5.05	5.04	5.05
112	138	5.27	5.1	5.31
113	139	5.34	5.11	5.35
114	140	5.09	4.93	5.01
115	141	5.04	4.89	4.96
116	142	5.11	4.93	4.97
117	143	5.4	5.26	5.43
118	144	5.34	4.98	5.39
119	145	5.34	5.11	5.33
120	146	5.32	5.15	5.27
121	147	4.9	5.26	4.98
122	149	5.1	5.26	5.18
123	152	5.03	5.23	5.01
124	156	5.36	5.22	5.32
125	161	6.12	5.81	6.04
126	164	6.16	5.67	6.07
127	167	5.49	5.24	5.48
128	170	5.53	5.14	5.47

129	172	5.11	4.96	5.08
130	173	5.13	5.09	5.2
131	175	5.03	4.99	5.01
132	176	5.25	5.26	5.21
133	177	5.07	5.06	5.04
134	178	5.08	4.97	5.02
135	179	5.15	4.86	5.09
136	180	5.27	5.18	5.22
137	181	5.3	5.26	5.35
138	182	5.2	5.05	5.18
139	183	5.09	5.04	5.06
140	184	5.26	5.11	5.25
141	185	5.41	5.28	5.4
142	186	5.45	5.24	5.43
143	187	5.54	5.39	5.55
144	191	4.92	5.02	4.95
145	192	5.35	5.1	5.41
146	193	4.79	5.02	4.63
147	194	4.94	5.16	4.8
148	195	5.23	5.11	5.23
149	196	5.05	4.72	5.14
150	197	5.2	5	5.31
151	199	5.03	5.04	4.92
152	200	5.41	5.23	5.44
153	201	5.16	5	5.1
154	203	5.09	5.22	5.14
155	204	3.43	6.19	3.38
156	205	3.53	5.66	3.74
157	206	2.81	5.49	2.91
158	207	2.84	5.85	3.02
159	208	3.02	5.62	3.15
160	209	2.66	5.75	2.76
161	210	3.31	4.94	3.58
162	211	3.2	5.07	3.42
163	212	3.33	5.77	3.41
164	213	3.74	5.42	3.69
165	214	3.4	5.22	3.53
166	215	3.43	5.07	3.55
167	218	4.76	4.86	4.82
168	221	3.26	6.12	3.25
169	222	3.29	5.82	3.34
170	223	3.58	6.21	3.53
171	224	3.19	6.24	3.24
172	225	3.38	6.11	3.26

173	226	3.22	6.29	3.23
174	227	2.96	6.6	2.8
175	228	2.91	6.4	2.87
176	229	3.18	6.18	3.08
177	230	3.77	4.85	3.96
178	231	3.46	5.25	3.63
179	232	3.11	5.51	3.31
180	233	3.88	4.99	4.04
181	234	3.22	5.21	3.43
182	236	3.06	6.28	3.15
183	237	2.99	6.56	2.88
184	238	4.79	4.94	4.75
185	239	4.7	5.08	4.66
186	240	4.93	5.2	4.87
187	241	4.92	5.06	4.95
188	242	3.1	5.58	3.23
189	243	3.08	5.74	3.22
190	244	3.22	5.91	3.33
191	245	2.7	5.97	2.86
192	246	2.64	5.89	2.84
193	247	2.7	5.93	2.91
194	248	3.76	5.22	3.75
195	249	3.23	5.65	3.37
196	250	3.32	5.3	3.4
197	251	3.26	5.73	3.3
198	252	3.67	5.57	3.7
199	253	3.85	5.18	3.89
200	254	3.62	5.45	3.72
201	255	3.44	5.27	3.53
202	256	3.2	5.3	3.31
203	257	3.08	5.85	3.24
204	258	3.14	5.72	3.2
205	259	3.18	5.51	3.3
206	260	3.89	5.23	3.97
207	261	3.41	5.32	3.56
208	262	3.54	5.39	3.66
209	263	3.84	5.1	3.88
210	264	3.8	5.09	3.95
211	265	3.16	6.26	3.25
212	266	3.4	5.99	3.31
213	267	2.7	6.74	2.62
214	268	2.43	6.56	2.44
215	269	2.28	6.68	2.26
216	270	2.68	5.91	2.73

217	271	2.46	6.26	2.37
218	272	2.22	6.34	2.34
219	273	1.94	6.71	1.98
220	274	2.92	6.26	2.98
221	275	3.09	5.88	3.32
222	276	3.84	5.55	3.85
223	277	2.77	6.26	2.97
224	278	2.68	6.45	2.86
225	279	3.71	5.82	3.75
226	280	2.54	6.13	2.73
227	281	2.33	5.62	2.65
228	282	2.48	5.33	2.89
229	283	3.98	5.64	4.02
230	284	3.66	5.89	3.66
231	285	3.58	5.22	3.91
232	287	4.05	4.91	3.94
233	288	2.82	5.78	3.08
234	289	3.59	6.45	3.46
235	290	4.1	5.26	3.97
236	291	2.66	6.34	2.65
237	292	3.19	5.84	3.05
238	293	3.48	5.68	3.45
239	294	2.85	5.9	3.13
240	295	2.54	6.6	2.64
241	296	2.66	6.68	2.64
242	297	2.76	6.35	2.64
243	298	3.96	4.81	4.23
244	299	3.26	5	3.49

Table S4. Internal consistency reliability for each type and dimension

	Valence	Arousal	Dominance
Total	0.98	0.99	0.98
Positive	0.99	0.99	0.99
Neutral	0.91	0.94	0.91
Negative	0.97	0.98	0.98

Table S5. Internal consistency reliability for each category and dimension

	Valence	Arousal	Dominance
People and human faces	0.91	0.96	0.92
Animals	0.93	0.97	0.93
Objects	0.80	0.88	0.80
Landscapes	0.93	0.94	0.94

Table S6. Criteria validity of short videos in four categories

	Valance	Arousal	Dominance
All videos	0.901^{**}	0.607^{**}	0.892^{**}
People and face	0.756^{**}	-0.092	0.770^{**}
Animal	0.567	-0.250	0.650
Object	0.601^{**}	0.451^{*}	0.515^{*}
Landscape	0.900^{*}	-0.684	0.900^{*}
